# Fighting the Cause of Alzheimer’s and GNE Myopathy

**DOI:** 10.3389/fnins.2018.00669

**Published:** 2018-10-15

**Authors:** Shreedarshanee Devi, Rashmi Yadav, Pratibha Chanana, Ranjana Arya

**Affiliations:** School of Biotechnology, Jawaharlal Nehru University, New Delhi, India

**Keywords:** amyloid β, NFT, GNE, hyposialylation, sialic acid, ER stress, apoptosis, autophagy

## Abstract

Age is the common risk factor for both neurodegenerative and neuromuscular diseases. Alzheimer disease (AD), a neurodegenerative disorder, causes dementia with age progression while GNE myopathy (GNEM), a neuromuscular disorder, causes muscle degeneration and loss of muscle motor movement with age. Individuals with mutations in presenilin or amyloid precursor protein (APP) gene develop AD while mutations in GNE (UDP *N*-acetylglucosamine 2 epimerase/*N*-acetyl Mannosamine kinase), key sialic acid biosynthesis enzyme, cause GNEM. Although GNEM is characterized with degeneration of muscle cells, it is shown to have similar disease hallmarks like aggregation of Aβ and accumulation of phosphorylated tau and other misfolded proteins in muscle cell similar to AD. Similar impairment in cellular functions have been reported in both disorders such as disruption of cytoskeletal network, changes in glycosylation pattern, mitochondrial dysfunction, oxidative stress, upregulation of chaperones, unfolded protein response in ER, autophagic vacuoles, cell death, and apoptosis. Interestingly, AD and GNEM are the two diseases with similar phenotypic condition affecting neuron and muscle, respectively, resulting in entirely different pathology. This review represents a comparative outlook of AD and GNEM that could lead to target common mechanism to find a plausible therapeutic for both the diseases.

## Introduction

Aging is the process, which initiates with subclinical changes at molecular level including accumulation of mutations, telomere attrition, epigenetic alterations resulting in genome instability (López-Otín et al., 2013). These changes multiply at a very fast rate, ultimately leading to the morphological and functional deterioration of brain by progressive loss of the neurons, reduction in the levels of neurotransmitters at the synaptic junction and disruption of integrity of the brain (Sibille, 2013). In addition to neurons, muscle cells are also affected with age. Loss of muscle mass, reduction in muscle fiber size and number is observed in muscles with age that decreases muscle strength (Narici and Maffulli, 2010; Siparsky et al., 2014). Thus, age is a common risk factor for both neurodegenerative and neuromuscular diseases, that progress with time.

The neurodegenerative disorders like Alzheimer’s disease (AD), Parkinson’s disease, Huntington’s disease and amyotrophic lateral sclerosis (ALS) share similar pattern of brain alterations and relate to each other at sub-cellular levels in numerous studies (Garden and La Spada, 2012; Montie and Durcan, 2013). Oxidative stress and altered Ca^2+^ and mitochondrial dysfunctions cause neuronal damage with age (Thibault et al., 1998, 2001). Further, neurons do not divide (with rare exceptions), thus cellular damage tend to accumulate with age (Sibille, 2013). Similarly neuromuscular disorders such as multiple sclerosis, muscular dystrophy, GNE related myopathy, Myasthenia gravis, Spinal muscular atrophy and ALS show subcellular damage in muscle cells where oxidative stress and altered calcium/mitochondrial, and ER stress are observed (Kanekura et al., 2009; Roussel et al., 2013; Stone and Lin, 2015; Xiang et al., 2017). Muscle cells are also among the least dividing cells with average lifespan of 15 years or sometimes reaching four decades. Due to its long life span like neurons, the cellular damage in muscle also accumulates in due course of time. As the age progresses, the satellite cells of muscle decline reducing the regeneration capacity of healthy muscle in place of affected cells (Narici and Maffulli, 2010). Whether there is any correlation of cellular damage in neurons versus muscle cells that can be a common therapeutic target is not known.

Indeed some disorders such as ALS can be placed in either of the two disorders as it affects both neurons and muscle cells. Several neuromuscular disorders, which include muscular dystrophies have reported degeneration of neurons in brain and affect the cognitive function leading to memory loss (Anderson et al., 2002; Ricotti et al., 2011). In ALS, loss of motor neurons affect the movement of various muscles of body leading to muscle wasting and paralysis, along with cognitive impairment (Taylor et al., 2016). Interestingly, a novel missense mutation (histidine to arginine at 705 amino acid) in GNE gene (UDP *N*-acetylglucosamine 2 epimerase/*N*-acetyl Mannosamine kinase) was observed in familial ALS patient (Köroğlu et al., 2017). Mutation in GNE gene causes GNEM, a rare neuromuscular disorder with completely different pathology compared to ALS (Huizing and Krasnewich, 2009). This raises a possibility of a missing link between the two disorders where the pathomechanisms might merge at a common target.

In this review, we have correlated and compared Alzheimer’s disease, a neurodegenerative disorder with GNEM, a neuromuscular disorder and put forth how these diseases share common pathological events like aggregation of misfolded proteins, oxidative stress, mitochondrial dysfunction, autophagy and cellular death. This will help us to find a common therapeutic approach for the treatment of these diseases.

## Epidemiology

Among various neurological disorders, Alzheimer’s disease is the most common form of dementia accounting for 60–80% of all the cases of dementia, with worldwide prevalence above 45 million^1^. It is more prevalent in the Western European and North American population. On the other hand, GNEM is a rare genetic neuromuscular disorder with worldwide prevalence of 1–9 in a millionth population (Orphanet^2^). GNEM has been reported in the Irish, Jewish, Japanese and Indian populations. Also, there are reports of GNEM from North America, European (United Kingdom and Scotland) and other Asian country like Thailand (Bhattacharya et al., 2018).

## Causes, Characteristics and Genetic Predisposition

AD is a multifactorial disease without any single cause. The main characteristic features of AD are senile plaques, composed mainly of extracellular amyloid-β (Aβ) peptides, and Neurofibrillary Tangles (NFTs) formed after accumulation of intracellular hyperphosphorylated tau (Serrano-Pozo et al., 2011). GNEM is caused by autosomal recessive mutation in GNE gene responsible for sialic acid biosynthesis. The characteristic features for GNEM involves weakness in the distal muscles, sparing the quadriceps, presence of rimmed vacuoles in muscle fibers and tubulofilamentous inclusions of aggregated proteins such as Aβ and phosphorylated tau (Jay et al., 2009). Despite the differences in tissues that are affected in the two diseases, accumulation of aggregates of amyloid-β and tau are common characteristics of both the diseases.

Initial symptom of AD is gradual loss in ability of the person to remember new information (Souchay and Moulin, 2009). The greatest risk factor for the development of AD is age as its pathological features increase exponentially with age (doubling every 5 years after the attainment of 65 years of age) (Querfurth and LaFerla, 2010). In GNEM, the initial symptoms include foot drop and weakness in the distal muscles, which gradually worsen with age toward wheel-chair dependence of patients. In GNEM, unlike AD, the brain function has been reported as normal (Anada et al., 2014). The onset of AD is late adulthood while GNEM onset is early adulthood during the second or third decade of life. How aging leads to sudden onset of GNEM is not known.

Beside aging, AD is caused due to mutation in either the presenilin genes or in Amyloid Precursor Protein (APP) gene (Goate et al., 1991; Hutton and Hardy, 1997; Holtzman et al., 2011). There is also an increased risk of AD in individuals suffering from Down’s syndrome because chromosome 21 includes a gene encoding the production of APP (Wiseman et al., 2015). The epsilon four allele of the apolipoprotein E gene (APOE) located on chromosome 19 is found to be a risk factor for AD (Reiman et al., 2005). People with a history of diabetes, hypertension, obesity, smoking, head injury leading to memory loss and a family history of AD in close relatives are at a greater risk of AD (Barnes and Yaffe, 2011). The prevalence of AD is higher in women and less educated masses (Letenneur et al., 2000).

On the other hand, GNEM is caused due to mutation in GNE (UDP-GlcNAc 2-epimerase/ManNAc kinase) gene that catalyzes the first two rate limiting steps in the biosynthesis of sialic acid (Jay et al., 2009). Whether hyposialylation is the only cause of GNEM is still unknown. GNEM is a genetic disorder and not known to be associated with lifestyle disease. No gender bias has been reported for GNEM. A complete comparison of characteristics of both AD and GNEM has been described in **Table 1**.

**Table 1:** Comparison of the characteristics of AD and GNEM.

	Characteristics of AD and GNEM	
	AD	GNEM
Disease type	Neurodegenerative	Neuromuscular
Onset of the disease	Early (5%) and late onset (>95%)	Early adulthood
Age	Mainly above 65 years of age	20–30 years of age
Prevalence	45 Million and above	∼1–9 in one Million
Demographics	Worldwide but common in Western Europe and North American population	Jewish, Japanese and Indian population
Initial signs	Loss in memory	Foot drop
Gender biasness	Higher in women	Not found
Mutation	FAD-autosomally dominant	Autosomal recessive
Genetic defects	Mutations in Presenilin genes, APOE gene	Mutations in GNE gene
Symptoms	Memory loss, agitation, sleeplessness, and delusions	Foot drop, weakness in distal muscles, and difficulty in walking
Progressiveness	Fast	Slow
Brain function	Affected	Not affected
Pathological effect	Damage to limbic system and neocortical region, Senile plaques and NFTs, aggregation of proteins	Rimmed vacuole of aggregated proteins tubulofilaments and small fibers, cytoplasmic and nuclear inclusion bodies, aggregation of proteins
Diagnosis	Medical history, pathological diagnosis of plaques and NFTs, MRI and CT scan of brain lesions, levels of serum B12, TSH, T4 etc.	Time of disease onset, Gait study, walking pattern, pathological study of rimmed vacuoles and other factors, biallelic mutations in GNE gene through sequencing
Treatment	Acetylcholinesterase (Ach esterase) inhibitors, drugs targeting Aβ and tau protein accumulation	Supplementation with sialic acid and its precursor molecules, IVIG administration, gene therapy

## Disease Pathology

In normal condition, neuronal cells release soluble Aβ after cleavage of a cell surface receptor called APP. In case of AD, the cleavage is abnormal leading to the precipitation of Aβ into dense beta sheets and formation of senile plaques (Zhang et al., 2011). To clear the amyloid aggregates, an inflammatory response is generated by astrocytes and microglia leading to the destruction of adjacent neurons and their neuritis (Norfray and Provenzale, 2004; Querfurth and LaFerla, 2010).

The tau protein is a microtubule stabilizing protein and has a role in intracellular transport (both axonal and vesicular). In its abnormally hyper-phosphorylated form, tau form intracellular aggregates called the NFTs or senile plaques, interfering with normal axonal transport of molecules along microtubules (Norfray and Provenzale, 2004).

In GNEM, main pathological feature includes formation of rimmed vacuoles, which is comprised of aggregated proteins such as Aβ and tau (Nalini et al., 2010). Cytoplasmic and nuclear inclusion bodies have also been observed by electron microscopy in muscle biopsies, which contain degradative products from the membrane, cytoplasmic tubulofilaments and mitochondria with irregular size and shape (Huizing and Krasnewich, 2009). However, since GNE is a key sialic acid biosynthetic enzyme, mutation in GNE affects the sialylation of proteins (Noguchi et al., 2004). The immunohistochemistry of GNEM muscle samples revealed upregulation of αβ-crystallin, NCAM, MHC-1, and iNOS levels (Fischer et al., 2013). NCAM was hyposialylated in GNEM and proposed as diagnostic marker for GNEM (Ricci et al., 2006). In aging brain and AD, the expression and function of NCAM and MHC-1 was altered that may result in synaptic and cognitive loss (Aisa et al., 2010). Also, reduced polysialated-NCAM load was reported in entorhinal cortex causing AD (Murray et al., 2016). Thus, NCAM sialylation can be a common target in the pathology of AD and GNEM in addition to Aβ and tau accumulation.

## Diagnosis

Medical and family history of individuals, which include psychiatric history, changes in behavior and cognitive functions, help in the diagnosis of AD. Amyloid plaques, presence of NFT’s and distribution in the brain are used to establish the disease by an autopsy based pathological evaluation. The clinical diagnosis of AD is about 70–90% accurate relative to the pathological diagnosis (Beach et al., 2012).

GNEM is clinically characterized by weakness in tibialis anterior muscles with a unique sparing of the quadriceps leading to foot drop, gait abnormalities, mild or no elevation in serum creatine kinase levels with no involvement of cardiac muscles, usually in the second or third decade of life (Nalini et al., 2013). Pathologically, GNEM is characterized by presence of rimmed vacuoles in muscle biopsies, without inflammation (Argov and Yarom, 1984). The confirmation of GNEM mainly relies on identification of bi-allelic mutation in GNE gene. As more than 190 mutations in GNE have been identified worldwide, complete sequencing of the GNE is necessary for diagnosis of GNE myopathy.

## Comparative Analysis of Molecular Mechanisms Affecting AD and GNEM

### Effect of Glycosylation, Particularly Sialylation, in AD and GNEM

Glycosylation is the process of incorporation of glycan, either monosaccharides or oligosaccharides, unit to proteins and lipid moieties (Spiro, 2002). The role of glycosylation in case of AD was first reported when impaired glucose metabolism increased toxicity from Aβ and affected glycosylation pattern (Ott et al., 1999; Peila et al., 2002; Chornenkyy et al., 2018). Several key proteins involved in Aβ deposition cascade such as APP, BACE-1 (β secretase), γ-secretase, nicastrin, neprisilin (NEP) undergo altered glycosylation in AD (Kizuka et al., 2017). Deletion of N-glycosylation of APP protein results in its reduced secretion (Schedin-Weiss et al., 2014). APP trafficking from trans-Golgi network to plasma membrane and non-amyloidogenic processing is enhanced by *O*-GlcNAcylation of APP (Chun et al., 2015). Interestingly, enhanced sialylation of APP increased APP secretion and Aβ production (Nakagawa et al., 2006). Defect in sialic acid biosynthesis due to mutation in GNE affects sialylation of glycoproteins in GNEM. Several proteins such as neural cell adhesion molecule (NCAM), α-dystroglycan, integrin, IGF-1R, and other proteins have been found with altered sialylation in absence of functional GNE (Huizing et al., 2004; Ricci et al., 2006; Grover and Arya, 2014; Singh et al., 2018). However, changes in glycosylation pattern of APP or Aβ are not studied in GNEM despite elevated levels of APP reported in ALS and GNEM (Koistinen et al., 2006; Fischer et al., 2013). Thus, there is a need to investigate whether hyposialylation of muscle cells, as effect of mutation in GNE, affects the glycosylation pattern and sialylation of accumulated glycoproteins and proteins like Aβ, presenilin-1 etc.

Proper glycosylation of nicastrin (a subunit of γ-secretase) affects its trafficking to Golgi apparatus and proper binding to presenilin-1, thereby, inhibiting APP processing and γ-secretase substrate preference (Yang et al., 2002; Xie et al., 2014; Moniruzzaman et al., 2018). Expression of glycosylated NEP, protein involved in Aβ clearance, is also reduced in AD (Reilly, 2001). Interestingly, in GNEM also, the glycosylation and sialylation of neprilysin is dramatically reduced, affecting its expression and normal enzymatic activity (Broccolini et al., 2008). The effect of reduced activity in NEP in GNEM may lead to its failure of clearance of Aβ from muscle. Additionally, it has also been reported that enzyme GNE undergoes *O*-GlcNAcylation thereby, modulating its enzymatic activity (Bennmann et al., 2016). Thus, it would be of interest to study effect of altered sialylation due to GNE mutation on glycosylation pattern of aggregating proteins.

Several reports indicate alteration of protein sialylation to be a leading cause of AD (Wang, 2009; Schnaar et al., 2014). Binding of Aβ to cells is sialic acid dependent as its binding to surface is mediated through sialylated gangliosides, glycolipids, and glycoproteins (Ariga et al., 2001). The levels of sialyltransferase reduce with age that may contribute to altered sialic acid levels (Maguire et al., 1994; Maguire and Breen, 1995). In addition, clearance of Aβ by microglia is enhanced in absence of sialylated immunoglobulin, CD33 (siglec-33) (Jiang et al., 2014; Siddiqui et al., 2017). This suggests that sialylation is important for Aβ uptake and accumulation.

Interestingly, altered levels of sialyltransferases ST3Gal5 and ST8Sia1 were reported in HEKAD293 cells overexpressing wild type recombinant GNE resulting in increased levels of gangliosides GM3 and GD3 (Wang et al., 2006). Thus, GNE may affect sialyltransferases with an unknown mechanism. Molecules affecting sialyltransferase levels may influence Aβ uptake in both GNEM as well as AD. Thus, changes in the sialylation pattern of Aβ deposition cascade proteins in muscle cells may affect rimmed vacuole formation in GNEM and offer new therapeutic approach.

### Role of Cytoskeleton Network in AD and GNEM

Cytoskeletal proteins are important functional proteins in both neuronal and muscle cells. In muscle, they help in conducting contraction and movement, while in neurons, they have a vital role in neuronal plasticity that is important for learning and memory process. Cytoskeletal proteins include different proteins like actin, tubulin, and lamin that provide mechanical support to the cell and modulate their dynamics inside the cell.

Tau, the first microtubule associated protein to be identified, was found to be one of the important hallmarks of AD along with Aβ. Tau directly helps in self-assembly of microtubule from tubulin. In AD, tau is found to be hyperphosphorylated at different site than normal (Gong et al., 2005; Hanger et al., 2007). The extent of tau aggregation is correlated with amount of phosphorylation at different sites (Iqbal et al., 2008). Also increased auto-antibodies of tubulin and tau were found in the serum of AD patients indicating a robust target for disease diagnosis (Salama et al., 2018). In GNEM, phosphorylated tau has been observed to accumulate in rimmed vacuoles (Nogalska et al., 2015), but whether aggregated tau is hyperphosphorylated from the normal form is not yet studied.

Actin dynamics and modulation of G-actin and F-actin is an important feature for neuronal plasticity and memory developments (Penzes and Rafalovich, 2012). Impaired cognitive function has been reported in AD pathology where cofilin-1, an actin depolymerizer, was found to be inactive (Barone et al., 2014). Inactivation of cofilin 1 contributes to actin dependent impairment of synaptic plasticity and thus, learning (Rust, 2015). Further, cofilin-1 inactivation is γ-secretase dependent, which controls Aβ peptide production. Also, cofilin-actin rods result in synaptic loss in AD (Bamburg et al., 2010). Small GTPases like RhoA, Rac1, and Cdc42 regulate APP, formation of Aβ and neurotoxicity (Boo et al., 2008; Wang et al., 2009). Phosphorylation of collapsin mediator response protein-2 (CRMP-2) in AD disrupts its binding with kinesin hampering axonal transport and resulting in neuronal defect (Mokhtar et al., 2018). RhoGTPases also play important role in muscle differentiation and muscle contraction (DeHart and Jones, 2004; Zhang et al., 2012). Interestingly, GNE has been shown to interact with CRMP-1, α-actinin-1, and α-actinin-2, key cytoskeletal regulatory proteins (Weidemann et al., 2006; Amsili et al., 2008; Harazi et al., 2017). Being an actin binding protein, binding of α-actinin-1 and α-actinin-2 with GNE raises a possibility of impaired actin function in GNEM. Differential cytoskeletal protein expression was observed in muscle biopsy samples of GNEM patients (Sela et al., 2011). Upstream of actin, FAK (focal adhesion complex) and integrin (extracellular matrix protein) function was affected in mutant GNE cells (Grover and Arya, 2014). It has also been reported that induction of Aβ led to the increased expression of FAK and autophosphorylation at Tyr397 (Han et al., 2013). However, role of RhoA, actin, cofilin needs to be further elucidated in GNEM. Taken together these studies indicate cytoskeletal proteins to be a common target that regulate Aβ production and need therapeutic intervention to explore effective molecules.

### Mitochondrial Dysfunction in AD and GNEM

Mitochondria are self-dividing organelles undergoing fission and fusion inside a cell. It is the power house of a cell that provides energy by oxidative phosphorylation during TCA cycle. Neurons and muscle cells have higher demand for mitochondria for their neuronal processes and muscle contraction, respectively. It has been reported that different cytoskeletal proteins help in motility of mitochondria in the cytoplasm (Lackner, 2013). Accumulation of Aβ and increased cellular death has been reported upon dissection of brains of AD patients (Cha et al., 2012). Further, Aβ accumulation in mitochondria precedes amyloid plaque, indicative of an early stage AD (Ankarcrona et al., 2010). In the early stages of AD, the number of mitochondria in the affected neurons is highly reduced leading to decreased glucose metabolism and impaired TCA cycle enzyme activity (Bubber et al., 2005; Mosconi, 2005). Additionally, elevated level of oxidative damage and significant increase in mutation of mtDNA and cytochrome c oxidase has been reported in AD patients (Castellani et al., 2002). Further, impaired mitochondrial trafficking has been observed in rat hippocampal neurons upon exposure to sub-cytotoxic levels of Aβ (Rui et al., 2006). Altered calcium homeostasis affects ATP generation and cause mitochondrial dysfunction (Supnet and Bezprozvanny, 2010; Swerdlow, 2018).

In GNEM, upregulation of a number of mitochondrial genes and transcript encoding mitochondrial proteins like COX, Cytochrome C Oxidase, ATPases, NADH dehydrogenase etc., have been reported in GNEM patient muscle biopsies (Eisenberg et al., 2008). Vacuolar and swollen mitochondria indicative of structure and functional dysfunction have been observed in HEK cells with mutated GNE (Eisenberg et al., 2008). Since function of mitochondria is dependent on its structure, increased branching of mitochondria observed in cells of GNEM patients could lead to oxidative stress (Eisenberg et al., 2008). Thus, both GNEM and AD show mitochondrial dysfunction. It would be of interest to determine the stage at which mitochondria are affected in GNEM and whether any Aβ accumulation occurs in mitochondria besides rimmed vacuoles.

In AD mouse study, COX gene knock out reduced oxidative stress by reducing Aβ plaque formation (Fukui et al., 2007). Inhibition of COX2 function results in protection of neurons and reduces the accumulation of Aβ in neurons of AD transgenic mice (Woodling et al., 2016). In GNEM, COX7A protein is reported to be upregulated (Eisenberg et al., 2008). Thus, inhibiting COX gene in GNEM may reduce mitochondrial oxidative stress and inhibit Aβ aggregate formation in GNE deficient cells and could serve as an important therapeutic target.

### Effect of Oxidative Stress in AD and GNEM

Oxidative stress is a key player in many neurodegenerative diseases. With age, oxidative stress in brain elevates due to imbalance of redox potential leading to generation of reactive oxygen species (ROS) (Andreyev et al., 2005; Wang and Michaelis, 2010). When the amount of ROS species produced is greater than scavenged by ROS defense mechanisms, it leads to oxidative stress leading to cell damage (Feng and Wang, 2012). Reports suggest that Aβ(1-42) accumulation is associated with oxidative stress in hippocampal neuron of *C. elegans* (Yatin et al., 1999). Phosphorylation of tau is also reported to be increased during oxidative stress via activation of glycogen synthase kinase 3-β (Lovell et al., 2004). Aberrant S-nitrosylation of proteins at cysteine residue of ApoE, Cdk5, and PDI leads to oxidative stress and neurodestruction (Zhao et al., 2014). In fact, oxidation of proteins in neurons that control Aβ solubilization and tau hyperphosphorylation severely affect progression of AD.

In GNEM, upregulation of cell stress molecules, such as Aβ oligomers, αβ-crystallin that signals to elevate APP protein was reported (Fischer et al., 2013). Upregulation of iNOS enzyme suggested that cell stress in GNE myopathy is mainly due to NO-related free radicals (Fischer et al., 2013). In GNEM patients and mouse model, proteins were found to be highly modified with S-nitrosylation (Cho et al., 2017). In AD, generation of NO correlates with the activation of iNOS in glial cells. Generation of NO by iNOS is robust and render neurotoxicity, contributing to neuronal death and injury (Zhao et al., 2014). Atrogenes and oxidative stress response proteins are highly upregulated in hyposialylated condition and supplementation with sialic acid restores ROS levels in muscle cells (Cho et al., 2017). Additionally, in HEK293 cell based model system for GNEM overexpressing pathologically relevant GNE mutation, PrdxIV, an ER resident Peroxiredoxin was found to be downregulated. The downregulation of Prdx IV may disturb the redox state of ER, affecting proper folding of proteins eventually leading to ER stress (Chanana et al., 2017). Also expression level of Prdx I and Prdx IV was substantially decreased in post-mortem brain of AD with higher level of protein oxidation (Majd and Power, 2018). These studies suggest that oxidative stress may be common to both the disorders. ER based peroxiredoxins may play an important role in the pathology of both the diseases.

### Role of Endoplasmic Reticulum and Chaperones in Protein Aggregation

Endoplasmic reticulum is an important cellular organelle involved in proper folding and processing of proteins. Perturbation in functioning of ER leads to misfolding of proteins and eventually protein aggregation, which is the key feature in several neurodegenerative diseases. Accumulation of misfolded proteins in ER elicits ER stress and unfolded protein response (UPR) that triggers cell death by apoptosis to eliminate cell toxicity (Tabas and Ron, 2011). Misfolded proteins that are retained in ER undergo proteosomal degradation via ER-associated degradation or ERAD (Smith et al., 2011). Activation of UPR proteins such as IRE1 and chaperone, GRP78, have been reported in the cortex and hippocampal tissue of AD brain (Hoozemans et al., 2005; Lee et al., 2010a). Activation of UPR proteins such as IRE1α, PERK, and ATF6 have been reported in AD by Xiang et al. (2017). Even GNEM muscle biopsies revealed upregulation of different UPR proteins including GRP78/BiP, GRP94, calnexin, and calreticulin, which are ER resident chaperones. The same study showed localization of GRP78/BiP and GRP94 with Aβ in the ER (Li et al., 2013). Upregulation of chaperone GRP94 is reported in HEK cell based model of GNEM (Grover and Arya, 2014). Since upregulation of chaperones is also observed in GNEM, they may play an important role in protein aggregate and subsequently rimmed vacuole formation. Thus, small molecules affecting chaperone activity to enhance proper protein folding and inhibition of protein aggregation offer a promising therapeutic approach for GNEM.

Interestingly, calreticulin, molecular chaperone that modulates Ca^2+^ homeostasis, is downregulated in cortical neurons of AD patients and used as negative biomarker for AD progression (Lin et al., 2014). Another study reported that calreticulin co-localizes with both Aβ and APP and helps in proper folding of Aβ (Johnson et al., 2001). Stemmer et al have showed that calreticulin bound directly with Presenilin and Nicastrin molecular component of γ-secretase, along with Aβ (Stemmer et al., 2013). The binding of calreticulin with γ-secretase may direct the proper binding and cleavage of APP to Aβ. Due to the downregulation of calreticulin in neurons, serum γ-secretase losses its proper cleaving activity leading to misfolded Aβ and accumulation in neurons. Altered calreticulin levels could affect protein folding in GNEM as calreticulin interact with phosphodiisomerase (PDI) to serve chaperone function in ER. PDI interacts with peroxiredoxin IV, which is downregulated in GNE deficient cells (Chanana et al., 2017). Thus, calreticulin may need further investigation towards its role as molecular chaperones in GNEM.

Heat Shock Proteins (HSPs) present in the cytosol also help protein to achieve native structure and avoid aggregation (Franklin et al., 2005; Paul and Mahanta, 2014). Elevated levels of HSP70 and HSP27 were found in brain tissues of AD patients (Perez et al., 1991; Renkawek et al., 1993). HSP70 has been reported to interfere with the secretory pathway of APP by binding to APP and reducing Aβ production. Along with HSP70, HSP90 has been shown to degrade Aβ oligomers and tau via the proteasome degradation pathway (Lu et al., 2014). Overexpression of HSP70 and HSP90 helps to maintain tau homeostasis and increases its solubility, thereby preventing aggregation (Petrucelli et al., 2004). Overexpression of the chaperones also prevents the activation of Caspases, which may lead to neuronal death due to accumulation of aggregated proteins (Sabirzhanov et al., 2012). Proteomic study on GNEM patient biopsies also indicates an increase in HSP70, Crystallin and HSPB1 levels (Sela et al., 2011). Thus, more intensive research is demanded to explore chaperones as therapeutic drug targets for GNEM that can reduce protein aggregation and inhibit rimmed vacuole formation.

### Autophagy in AD and GNEM

Autophagy is the major degradative pathway for recycling of various proteins and organelles inside the cell, as it is essential for maintaining a balance between protein synthesis and degradation (Yang et al., 2009). Autophagy has been reported to be elevated when cells sense any kind of stress (Kwang et al., 2008). In AD, number of autophagosomes increase indicative of impaired recycling of cellular constituents (Funderburk et al., 2010). Mutation in Presenilin-1 gene affects lysosome mediated autophagy, reduces p62 protein levels leading to imbalance in tau proteostasis (Chui et al., 1999; Lee et al., 2010b; Tung et al., 2014). Many genes common to autophagy and AD pathology have been identified such as autophagy-related 7 (ATG7), BCL2, Beclin 1 (BECN1/ATG6), cyclin dependent kinase 5 (CDK5), Cathepsin D (CTSD), microtubule associated protein tau (MAPT/TAU), Presenilin-1, α-Synuclein (SNCA/PARK1/NACP), Ubiquitin 1 etc., (Uddin et al., 2018). Aβ accumulated intracellularly also regulates autophagy (Son et al., 2012). Tau pro-aggregates act as targets for macrophagy and chaperone mediated autophagy (Zare-Shahabadi et al., 2015).

**FIGURE 1:**
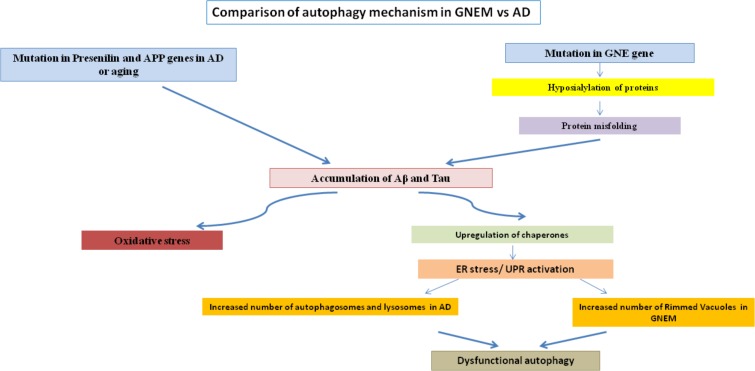
Comparison of autophagy mechanism in AD and GNEM. Mutation in genes of Presenilin and APP for AD and GNE for GNEM leads to the accumulation of various proteins like Aβ and tau. This accumulation causes oxidative stress and ER stress/UPR activation due to upregulation of chaperones, ultimately leading to dysfunctional autophagy as the number of autophagosomes, lysosomes, and rimmed vacuoles increases.

Rimmed vacuoles observed in GNEM pathology are also defined as clusters of autophagic vacuoles and multi-lamellar bodies, which contain congophilic amyloid proteins, ubiquitin and tau proteins (Nonaka et al., 2005). Higher expression of lysosomal-associated membrane proteins (LAMPs), LC3 and various other lysosomal proteins involved in autophagic pathway were observed in the skeletal muscle of the mice model for GNEM (Malicdan et al., 2007). Differential regulation of BCL2 in GNEM also supports that some common proteins of autophagy pathway in AD may play a role in GNEM autophagic vacuole formation. A comparison of the autophagic mechanisms in AD vs. GNEM is shown in **Figure 1**. Thus, it would be of interest to study and identify novel targets causing autophagy in GNEM and several autophagy stimulating drugs for AD may serve as therapeutic option for myopathy.

### Cell Death and Apoptosis

Cell death is the most common feature of the neurodegenerative diseases and occurs massively. In AD, neuronal loss is mainly in cerebral cortex and limbic lobe (Alzheimer’s Association, 2017). There are two major pathways for apoptosis, extrinsic pathway and intrinsic pathway. The extrinsic pathway involves cell surface receptors like TNF in which the binding of Aβ or Aβ oligomers to these receptors remains to be established but the pattern of activation of downstream Caspases (e.g., Caspases 2 and 8) involved in the extrinsic pathway is mediated by Aβ (Ghavami et al., 2014). In the intrinsic pathway, Aβ plays an important role as its intracellular accumulation in the ER cause ER stress and when it binding to a mitochondrial alcohol dehydrogenase leads to mitochondrial stress followed by activation of the downstream apoptotic markers (Lustbader et al., 2004). The upstream mediators of the apoptotic processes are yet to be determined, but the Caspases are activated in the process, which cleaves the tau protein leading to NFT formation (Dickson, 2004). Therefore, in AD, proteolysis of both APP and tau takes place leading to abnormal proteins, which aggregate and form lesions of fibrils extracellularly and intracellularly. Thus, direct involvement of Caspases in apoptosis of neurons is not yet established but many Caspases have been found to play a role in regulation of neuronal death upon Aβ accumulation (Behl, 2000; Dickson, 2004). Aβ(1-42) exposure leads to down regulation of anti-apoptotic proteins like Bcl-2 and upregulation of pro-apoptotic proteins like Bax, cytochrome-c and cleaved caspases in PC12 cells (Chen et al., 2018). Altered levels of various microRNAs that target neuropathological mechanisms have been reported in AD (Ma et al., 2017; Dehghani et al., 2018). Activation of programmed necrosis leading to cell death is reported in the brain of AD patients (Caccamo et al., 2017). The suppression of apoptotic cell signaling pathway proteins such as p38 MAPK can rescue tau pathology in AD (Maphis et al., 2016). These study suggest that various effector molecules targeting signaling proteins in the apoptotic pathway can play a role is preventing cell apoptosis caused due to Aβ accumulation or tau dysfunction and hence potential drug molecules for AD.

In GNEM, degeneration is seen in the myofibrils of the patient muscle biopsies, which might lead to rimmed vacuole formation (Yan et al., 2001). Similar to AD, activation of Caspases 3 and 9 was observed in the myoblast cells of the GNEM patient with M743T kinase mutation (Amsili et al., 2007). Along with this, increased pAKT levels was observed which suggests impairment in the apoptotic event (Amsili et al., 2007). Mitochondrial dependent apoptosis and disruption in both the structure and function of the mitochondria was observed in HEK cell based model system of GNEM over-expressing pathologically relevant GNE mutation (Singh and Arya, 2016). Also, activation of PTEN and PDK1 was observed in the myoblasts which might lead to muscle loss and on stimulation with insulin, activates PI3K and downstream signaling through AKT causing the activation of cell survival pathway (Harazi et al., 2014). Increased Anoikis, apoptosis due to loss of anchorage to extracellular matrix, was observed in pancreatic carcinoma cells when the GNE gene was silenced. Additionally, the level of CHOP has been reported to increase in GNE deficient cells indicative of apoptosis through ATF4-ATF3-CHOP pathway (Kemmner et al., 2012). Increased apoptosis due to internalization of Aβ peptides in hyposialylated C2C12 myotubes and skeletal muscles was observed in the patients of GNEM (Bosch-Morató et al., 2016). This suggests that sialylation has a role in Aβ uptake and cell apoptosis and molecules involved in apoptotic pathway can be therapeutic targets. Thus, molecular and cellular phenomenon for apoptosis in AD and GNEM seem to overlap despite difference in cell types, neuron vs. muscle cell, respectively.

**FIGURE 2:**
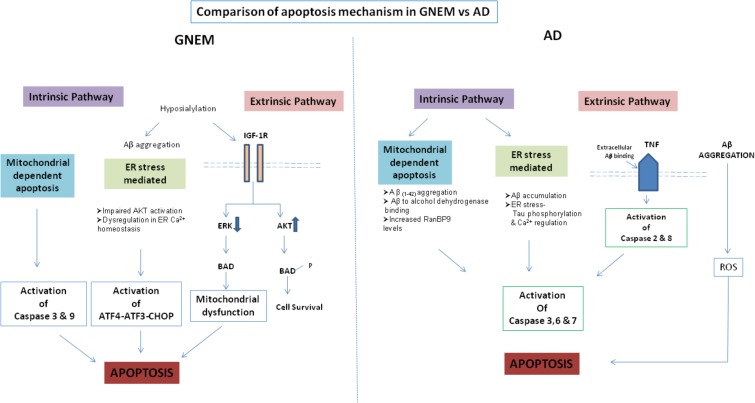
Comparison of apoptosis mechanism in AD and GNEM. In GNEM, intrinsic pathway is mediated through mitochondria as a consequence of mitochondrial dysfunction and consequent release of cytochrome C and activation of executioner caspases-Caspase-3 and Caspase-9. Further, in the extrinsic pathway, mutation in GNE causes hyposialylation of Aβ leading to its aggregation progressing toward ER stress where AKT pathway is impaired and sarcoplasmic calcium is released into the cytoplasm, eventually leading to apoptosis. In the extrinsic pathway, hyposialylation of IGF1R receptor leads to impairment of ERK pathway and activation of BAD, inhibiting anti-apoptotic Bcl-2 and thus leading to apoptosis. Whereas in AD, in the intrinsic pathway, Aβ accumulation and tau phosphorylation in the ER leads to ER stress. In the mitochondria of AD patients, Aβ binds to Aβ binding-alcohol dehydrogenase (ABAD) leading to mitochondrial dysfunction. Both ER and mitochondrial stress lead to activation of effector caspases 3, 6, and 7, which might cleave tau leading to formation of NFTs. In the extrinsic pathway, accumulated Aβ binds to ligand TNF leading to activation of Caspase 2 and 8.

A comparison of the apoptotic mechanisms in AD vs. GNEM has been described in **Figure 2**. Interestingly, treatment of GNE deficient cells with Insulin Growth Factor seems to rescue the apoptotic phenotype and hence could be a potential therapeutic target that counters apoptotic cell toward cell survival (Singh et al., 2018). In summary, proteins and drug molecules that rescue cell death phenomenon in AD by targeting common proteins, can be explored for GNEM therapy.

A complete comparison of molecular and cellular changes in AD and GNEM are listed in **Table 2**.

**Table 2:** Comparison of the molecular and cellular changes in AD and GNEM.

	Molecular and cellular changes in AD and GNEM	
	AD	GNEM
Glycosylation	• Impaired glucose metabolism • Glycosylation of proteins affected	• Glycosylation of NCAM, integrin, α-dystroglycan, IGF1R, neprilysin
Sialic acid involvement	Sialic acid dependent binding of Aβ to cells • Decrease Level sialyltransferases	Low Sialic acid production • Hyposialylation of glycoproteins.
Mitochondrial dysfunction	• Mitochondria number reduced • Aβ accumulation in mitochondria • Impaired TCA cycle • Mutation in mtDNA and cytochrome c oxidase • Impaired mitochondrial trafficking	• Vacuolar and swollen mitochondria • Increased branching of mitochondria • Upregulation of mitochondrial proteins like COX, Cytochrome C Oxidase, ATPases, NADH dehydrogenase
Oxidative stress	• Neurotoxicity and protein oxidation due to accumulated Aβ • Increased p-Tau • S-nitrosylation of different proteins like Cdk5, PDI, ApoE	• Upregulation of cell stress molecule • PrdxIV downregulated • Proteins found to be highly S-nitrosylated
ER stress	• XBP1 mRNA splicing leading to activation of IRE1α • GRP78/BiP, GRP94, calnexin and calreticulin upregulated • Co-localization of GRP78/BiP GRP94 and calreticulin with Aβ • Calreticulin binds with presenilin and neprisilin	• Upregulation of different UPR pathway proteins such as GRP78, GRP94, Calreticulin and Calnexin
Protein aggregation	• Aβ and p-tau proteins	• β-amyloid, phosphorylated Tau, TDP-43, α-synuclein
Chaperone involvement	• HSE (Heat Shock Element) associated with APP gene promoter • Elevated levels of HSP70 and HSP27 • HSP90 and HSP70 degrades Aβ oligomers and tau • Higher levels of HSP70 and HSP90 promotes the binding of tau to the microtubules	• Upregulation of various chaperones • The mutant protein preferentially retained in the ER
Apoptosis	• Activation of downstream caspases mediated by Aβ • Caspases cleaves the Tau protein • Nuclear chromatin clumping and apoptotic bodies	• Degeneration in myofibrils • Activation of Caspases 3 and 9 • Mitochondrial dependent apoptosis and disruption of mitochondria • Increased Anoikis • Increased levels of CHOP
Autophagy	• Failure of autophagy • Increased number of autophagosomes and lysosomes • Rab7 and LAMP proteins dysregulated	• Rimmed vacuoles-clusters of the autophagic vacuoles and multi-lamellar bodies • Higher expression of lysosomal-associated membrane proteins (LAMPs), LC3
**Inflammation**	**Occurs**	**Occurs rarely**
Cytoskeleton framework	• Aggregation of hyperphosphorylated tau • Inactivation of cofilin 1, SSH1 • Rac1 leads to APP accumulation • RhoA increases Aβ • Cofilin-actin rod results in synaptic loss	• GNE interact with Collapsin Response Mediator Protein-1 (CRMP-1), α-actinin-1 and α-actinin-2 • β-integrin mediated cell adhesion affected • Aβ induces FAK

## Treatment

There is no cure for AD till date as the medications available only help to control the symptoms of AD. The AD drug therapy includes drugs, which target neurotransmitter system of the brain such as Acetylcholinesterase (Ach esterase) inhibitors that increases neurotransmitter levels at synaptic junctions (Schenk et al., 2012). Three FDA approved acetylcholinesterase inhibitors are Rivastigmine, Galantamine (for mild AD), and Donepezil (for all stages of AD) are available (Schenk et al., 2012). Also Memantine, antagonist for *N*-methyl-D-aspartate (NMDA) receptor is used in combination with Ach esterase inhibitor. None of the pharmacological drugs are able to stop the damage and destruction of the neurons therefore, making the disease fatal.

Since, Aβ accumulation is one of the major causes leading to the disease; therefore drugs, which can lower the amount of Aβ accumulation in the brain are of prime importance. Secretase inhibitor drugs, inhibit the cleavage of APP into Aβ, therefore minimizing their accumulation (Imbimbo and Giardina, 2011). Another set of drugs used as a passive vaccination strategy in the form of antibodies, help in the clearance of Aβ species (Schenk et al., 2012). Several drugs were developed which completed Phase-III clinical trials but failed to demonstrate their efficacy in patients. The passive vaccination strategy in case of tau also proved to be ineffective (Wischik et al., 2014). A major limitation with respect to effectiveness of anti-amyloid drugs was thought to be late diagnosis of the disease. Thus, research focussing on the stage of initiation of amyloid formation could offer better drug targets. Indeed aducanumab, human monoclonal antibody, selective for aggregated form of Aβ showed reduced amyloid uptake and improved cognitive function in early AD patients (Scheltens et al., 2016).

In case of GNEM also, there is no treatment therapy available, which could reverse disease progression and stop muscle degeneration. Administration of *N*-acetylmannosamine, neuraminic acid, and sialyllactose in the mouse models of GNEM improved survival of the mouse by reduction in rimmed vacuole formation and β-amyloid deposition (Yonekawa et al., 2014). Gene therapy by administration of GNE gene lipoplex through intravenous infusion to the patients leads to an improvement in muscle strength and increased cell surface sialylation (Nemunaitis et al., 2010). An FDA approved molecular chaperone aiding in protein folding – 4-PBA (4-phenyl butyrate) has been proposed for GNEM (Krause, 2015). Anti-ActII activin antibody (bimagrumab or BYM338), an atrophic protein has been found to be helpful in preventing muscle atrophy in GNEM (Krause, 2015). Some of the compounds are under clinical trials such as sialic acid precursor, *N*-acetylmannosamine (ManNAc), and extended release sialic acid form, aceneuramic acid. However, due to lack of statistical significance in the cohort of patient study, the compound was discontinued by Ultragenyx (Mori-Yoshimura and Nishino, 2015; Argov et al., 2016).

Recent studies in GNEM indicate that sialic acid supplementation alone may not be sufficient to rescue disease phenotype. As discussed above several other cellular phenomena affect GNEM including accumulation of aggregated proteins such as β-amyloid and tau proteins. Sialic acid has been shown to affect β-amyloid uptake in C2C12 myoblast indicating role of sialic acid in β-amyloid uptake (Bosch-Morató et al., 2016). Thus, drug molecules affecting β-amyloid uptake and initiation of Aβ accumulation may serve as better therapeutic targets and offer common mechanism for AD as well as GNEM.

## Conclusion

While much is known for AD, GNEM is poorly understood rare disease. Lack of number of patient samples for GNEM also limits the study. Also, absence of appropriate animal model system for GNEM, as GNE^−/−^ mice are embryonically lethal at day E8.5, restricts the understanding for genotype to phenotype co-relation. There could be some interesting leads from AD studies that could help explore GNEM pathomechanism. While both diseases have lot of similarities at cellular level such as Aβ amyloid deposition, protein aggregation, autophagic vacuoles, major difference is that in AD, brain/neurons are affected while in GNEM, only muscles in particular anterior tibialis muscle cells are affected. No changes in the neurons of GNEM patients are reported. It would be of interest to study the stage of Aβ deposition in GNE deficient cells and whether protein aggregation could be prevented to slow the disease progression for GNEM. Also, whether there is any genetic predisposition of AD or GNEM in patient families would be important to understand epigenetics of these neurodegenerative disorders. Future studies could be planned toward deciphering common therapeutic targets for these disorders.

## Author Contributions

SD and RY have written the first draft of the manuscript. PC and RA revised and improved the first draft. RY prepared the tables and **Figure 2**. PC prepared **Figure 1**. RA edited and finalized the version. All authors have seen and agreed on the finally submitted version of the manuscript.

## Conflict of Interest Statement

The authors declare that the research was conducted in the absence of any commercial or financial relationships that could be construed as a potential conflict of interest.

## Footnotes

1 www.alzheimers.net

2 http://www.orpha.net/

## Abbreviations

Aβ: β amyloid
AD: Alzheimer’s disease
GNE: UDP-GlcNAc 2-epimerase/ManNAc kinase
GNEM: GNE myopathy
NFT: neurofibrillary tangles.
